# Modulation of Experimental Herpes Encephalitis-Associated Neurotoxicity through Sulforaphane Treatment

**DOI:** 10.1371/journal.pone.0036216

**Published:** 2012-04-27

**Authors:** Scott J. Schachtele, Shuxian Hu, James R. Lokensgard

**Affiliations:** Center for Infectious Diseases and Microbiology Translational Research, Department of Medicine, University of Minnesota, Minneapolis, Minnesota, United States of America; Consejo Superior de Investigaciones Cientificas, Spain

## Abstract

Reactive oxygen species (ROS) produced by brain-infiltrating macrophages and neutrophils, as well as resident microglia, are pivotal to pathogen clearance during viral brain infection. However, unchecked free radical generation is also responsible for damage to and cytotoxicity of critical host tissue bystander to primary infection. These unwanted effects of excessive ROS are combated by local cellular production of antioxidant enzymes, including heme oxygenase-1 (HO-1) and glutathione peroxidase 1 (Gpx1). In this study, we showed that experimental murine herpes encephalitis triggered robust ROS production, as well as an opposing upregulation of the antioxidants HO-1 and Gpx1. This antioxidant response was insufficient to prevent tissue damage, neurotoxicity, and mortality associated with viral brain infection. Previous studies corroborate our data supporting astrocytes as the major antioxidant producer in brain cell cultures exposed to HSV-1 stimulated microglia. We hypothesized that stimulating opposing antioxidative responses in astrocytes, as well as neurons, would mitigate the effects of ROS-mediated neurotoxicity both *in vitro* and during viral brain infection *in vivo*. Here, we demonstrate that the addition of sulforaphane, a potent stimulator of antioxidant responses, enhanced HO-1 and Gpx1 expression in astrocytes through the activation of nuclear factor-E2-related factor 2 (Nrf2). Additionally, sulforaphane treatment was found to be effective in reducing neurotoxicity associated with HSV-stimulated microglial ROS production. Finally, intraperitoneal injections of sulforaphane into mice during active HSV infection reduced neuroinflammation via a decrease in brain-infiltrating leukocytes, macrophage- and neutrophil-produced ROS, and MHCII-positive, activated microglia. These data support a key role for astrocyte-produced antioxidants in modulating oxidative stress and neuronal damage in response to viral infection.

## Introduction

The first line in defense of the brain against invading viral pathogens is the activation of local microglia and the recruitment of peripheral leukocytes, including macrophages and neutrophils. These cells work to eliminate the invading pathogen through the rapid and robust production of reactive oxygen species (ROS). Some of the reactive species generated include superoxide (O^2−^), hydroxyl radical (OH), hydrogen peroxide (H_2_O_2_), hypochlorite (OCl^−^), and peroxynitrite (OONO^−^). ROS production, while beneficial in clearing invading pathogens, can also cause irreparable harm through oxidative damage to crucial host cells. ROS have been implicated in toxicity associated with diverse neurodegenerative diseases such as Alzheimer's disease, Parkinson's disease, and Amyotrophic lateral sclerosis [1], [2]. Similarly, ROS production and its associated oxidative tissue damage contribute to herpes simplex virus (HSV)-1-induced neuropathology [3], [4], [5].

The brain employs various defense mechanisms to combat free-radical mediated oxidative tissue damage and subsequent neurotoxicity, including the upregulation of phase II antioxidant enzymes. Antioxidants are important because they directly remove reactive species from the extracellular matrix limiting free radical exposure to healthy tissues. Cellular oxidative stress triggers antioxidant production through nuclear translocation of the oxidative stress-induced transcription factor, nuclear factor-E2-related factor 2 (Nrf2). Nrf2 binds to an antioxidant response element (ARE) to induce transcription of multiple antioxidant genes, including heme oxygenase-1 (HO-1) and glutathione peroxidase-1 (Gpx1) [6]. Increased antioxidant enzyme expression has been shown to decrease neurotoxicity in models of ischemia and HIV-associated neurocognitive disorders [7], [8], [9]. Thus, modulation of antioxidant responses is a promising strategy for preventing secondary tissue damage from free-radicals during viral brain infection.

Sulforaphane (1-isothiocyanato-4-methylsulfinylbutane; SFN), an isothiocyanate that is naturally found in cruciferous vegetables, is a potent inducer of cellular antioxidants. SFN acts through the Nrf2/ARE pathway to enhance production of antioxidant tripeptides (glutathione; GSH) via the upregulation of the modifier and catalytic subunits of glutamate cysteine ligase GCLM and GCLC, respectively.[10], as well as by inducing transcription of free-radical neutralizing enzymes, including HO-1 and Gpx1 [11]. *In vitro* studies indicate that SFN can reduce ROS production in BV2 cells, a microglial-like cell line, as well as down-regulate macrophage activation [12]. In addition, SFN is an excellent candidate to modulate the antioxidant response during brain inflammation because of its ability to permeate the blood-brain barrier [12]. Indeed, peripheral injection of SFN into mice has been shown to confer anti-inflammatory and neuroprotective action in models of neurodegenerative diseases [13] and pathogen-induced brain inflammation [12].

HSV-1 infection of the brain results in devastating necrotizing encephalitis. Using a murine model of herpes encephalitis we have shown that intranasal delivery of HSV triggers a robust immune response, which includes the activation of resident microglial cells, infiltration of leukocytes, production of proinflammatory mediators, and focal tissue damage which, if left untreated, can result in prolonged neuroinflammation and compromised brain function or death [5], [14], [15], [16],[17]. It is becoming increasingly clear that ROS contribute to the secondary tissue damage that occurs during and subsequent to viral brain infection [18], [19]. Biproducts of oxidative tissue damage, including 8-isoprostane and 8-hydroxydeoxyguanosine, have been detected both during active herpes encephalitis as well as during latent herpes infection [5], [19]. These studies indicate that the neurotoxic effects observed during herpes encephalitis may not be simply due to viral replication, but may also result from secondary tissue damage originating from host-generated ROS.

In this study, we hypothesized that ROS-mediated neurotoxicity associated with experimental herpes encephalitis can be modulated by stimulating opposing antioxidative responses. *In vivo* analysis of ROS in infected mice confirmed the presence of significant oxidative stress during the peak of viral infection. Despite a concomitant increase in antioxidant enzyme mRNA expression *in vivo*, infected mice still amass significant oxidative tissue damage. Treatment of both neural cultures and purified astrocytes with SFN *in vitro* resulted in the robust expression of antioxidants which, subsequently, conferred protection to neurons upon exposure to HSV-stimulated, ROS-producing microglia. Furthermore, we show that systemic administration of SFN reduced macrophage and neutrophil brain infiltration, ROS production, and microglial cell activation *in vivo* during viral encephalitis.

## Results

### Robust ROS production and antioxidant gene induction during herpes encephalitis

**Figure 1:**
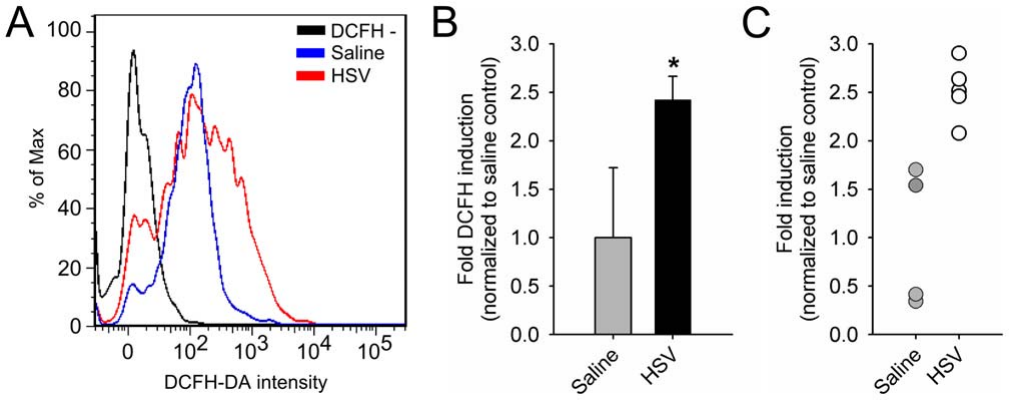
Increased brain ROS levels during herpes encephalitis. Balb/c mice were infected intranasally with 2×10^5^ PFU of HSV-1 strain 17 syn+ (n = 10). An equal volume of saline was delivered to control mice (n = 8). At 7 d p.i. whole brains were pooled, mononuclear cells were isolated and analyzed via flow cytometry using fluorescent-conjugated antibodies, CD11b-APC and CD45-APC-Cy7. CD11b^+^, CD45^hi^ macrophages/neutrophils were gated for further analysis of intracellular ROS via detection of DCFH-DA (20 µM). **A**) DCFH-DA fluorescence spectrum in CD11b^+^, CD45^hi^ cells from saline (blue) and HSV-infected (red) mice. Non-DCFH-loaded control is black. Composite (**B**) and individual (**C**) ROS data are presented as fold induction of HSV-infected mice (n = 5) over controls (n = 5). *p<0.05.

We have previously shown that herpes encephalitic mice exhibit increased accumulation of oxidative tissue damage biproducts [5]. Direct monitoring of *in vivo* ROS to establish the presence of elevated free radicals in the brains of HSV-1 infected mice has not been performed, but is essential to confirm the role of oxidative stress on herpes encephalitis-associated pathology. Using conjugated antibodies for CD11b and CD45, we have established a flow cytometry antibody staining regiment for the separation of brain infiltrating leukocytes (macrophages/neutrophils (CD11b^+^, CD45^hi^) and brain-resident microglia (CD11b^+^, CD45^int^) [5]. This, in combination with 2′,7′-Dichlorofluorescein diacetate (DCFH-DA), a fluorescent indicator of intracellular ROS, enabled quantification of free radical production by brain infiltrating monocytes during viral infection. Confirming our previous studies, we found that HSV-1 infection resulted in the robust migration of CD45+,CD11b^hi^ macrophages/neutrophils into the brain at 7 d post-infection (p.i.). At this time point post-infection, the majority of CD11b^+^, CD45^hi^ cells are macrophages, with <10% being neutrophils [5]. Analysis of CD45+,CD11b^hi^ macrophages/neutrophils for DCFH-DA fluorescence revealed a significant increase in ROS production in the brains of HSV-1 infected mice at 7 d p.i. compared to saline-infected controls (Figure 1).

**Figure 2:**
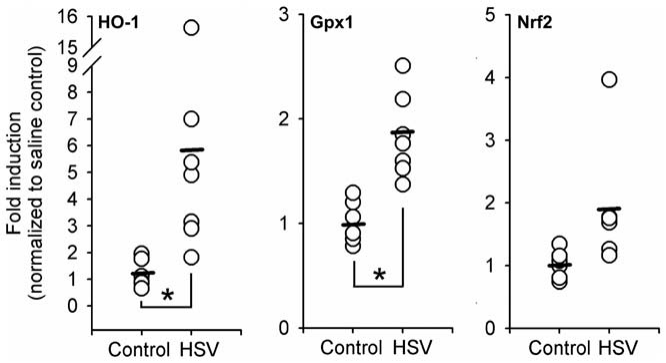
Antioxidant gene expression is upregulated in the brains of mice with herpes encephalitis. Balb/c mice were infected intranasally with 2×10^5^ PFU of HSV-1 strain 17 syn+ (n = 7). An equal volume of saline was delivered to control mice (n = 6). At 7 d p.i. mice were anaesthetized, dissected and 2 mm-thick sections of subcortical brain tissue were collected for mRNA extraction, cDNA synthesis and semi-quantitiative PCR analysis of HO-1 (*p = 0.01), Gpx1 (*p = 0.001), and Nrf2 expression.

We next investigated whether increased ROS levels in the brain of herpes encephalitic mice were associated with concomitant upregulation of a combatant and opposing antioxidative stress response. Using semi-quantitative, real-time (RT)-PCR, we probed Nrf2 as well HO-1 and Gpx1, two prototypical Nrf2-transcribed antioxidant proteins that exhibit neuroprotective capabilities during brain inflammation [13], [20], for changes in mRNA expression. In the subcortex of HSV-1 infected mice, we detected significantly elevated gene expression of both HO-1 (*p = 0.01) and Gpx1 (*p = 0.001) at 7 d p.i., while Nrf2 gene expression was not elevated compared to control mice (Figure 2).

### Astrocytes mediate antioxidative stress responses induced by HSV-1 stimulated murine microglia

**Figure 3:**
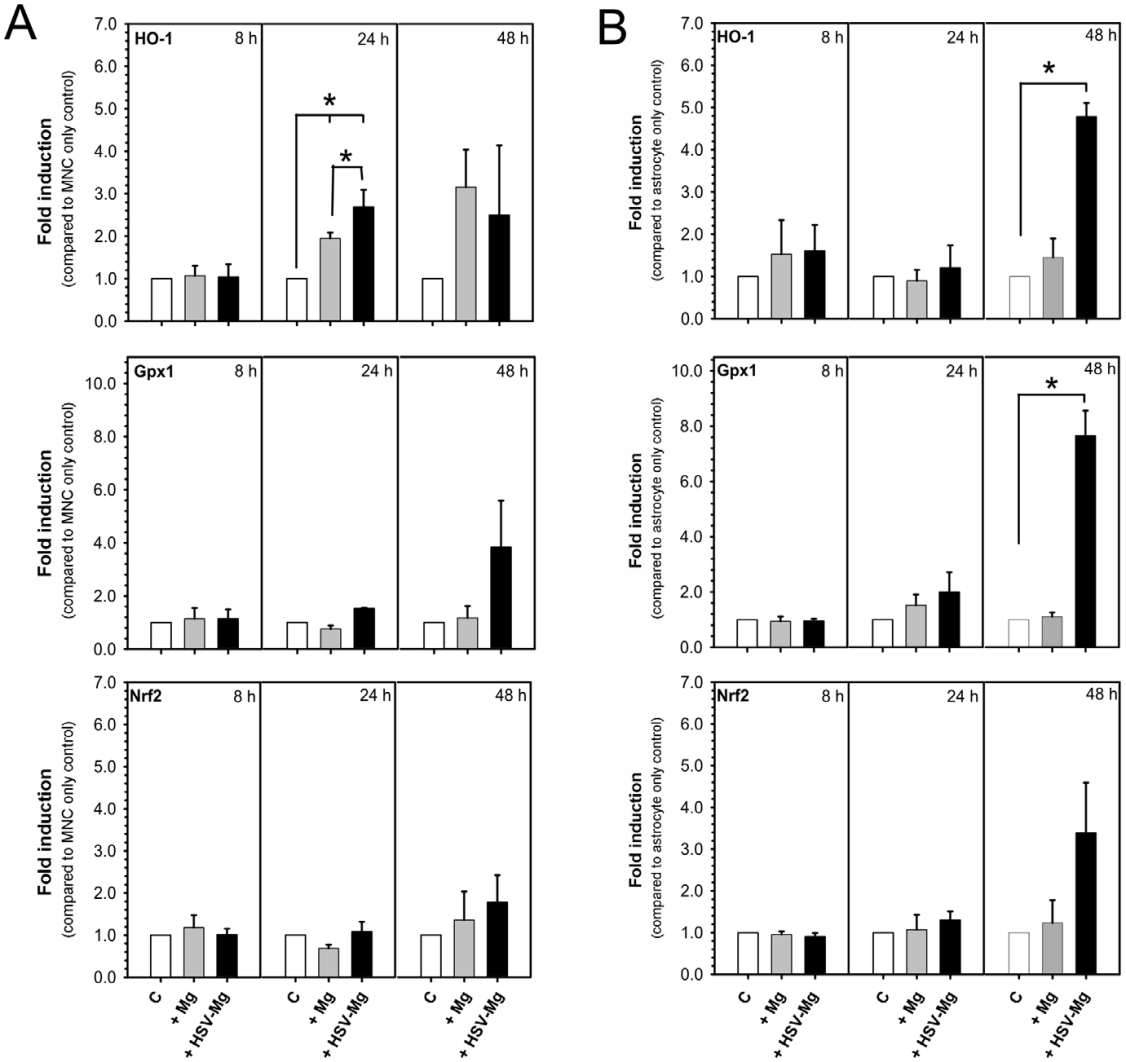
HSV-stimulated murine microglia induce antioxidant gene expression in purified mouse astrocytes cultures. Purified murine microglia obtained from neonatal Balb/c mice were infected with HSV-1 at 2 MOI for 90 min (+HSV−Mg) and added at a 1 to 5 microglia: neural cell ratio to either (**A**) murine mixed neural cultures (consisting of neurons, astrocytes and microglia; MNC) or (**B**) purified murine astrocyte cultures. Cultures were maintained for 8, 24 or 48 h before cell collection and RNA extraction. Unstimulated microglia (+Mg) and MNC or astrocyte only cultures (C) were used as controls. Data are presented as the mean fold induction ± standard error of the mean over saline controls (n = 3). Statistical significance was tested using an ANOVA single factor with PLSD Post-hoc analysis (StatView 5).

Exposure of cultured microglia to HSV-1 results in their activation, including production of neuroinflammatory cytokines, chemokines and ROS [3], [14]. HSV quickly induces robust microglial intracellular ROS production and, when co-cultured with mixed neural cultures (MNCs), consisting of approximately 85–90% neurons, 10–15% astrocytes and <2% microglia, results in the accumulation of significant oxidative damage and neurotoxicity after 48 h. Using this co-culture model in conjunction with semi-quantitative RT-PCR, we investigated the ability of HSV-stimulated microglial ROS to induce an antioxidant response in both MNC and purified murine astrocytes. We found that the addition of HSV-stimulated microglia (1∶5 microglia: neural cell ratio) did not result in a significant upregulation of Nrf2 or Gpx1 by 48 h post-incubation (Figure 3A). A small but significant transient upregulation of HO-1 mRNA was observed at 24 h post-incubation in MNC treated with either unstimulated (p≤0.05) or HSV-stimulated microglia (p≤0.001). This correlated with higher HO-1 expression in cultures with HSV-stimulated microglia than those receiving unstimulated microglia (p≤0.05).

Astrocyte-specific upregulation of antioxidants has been identified as a preferential pathway for protection in the face of disease-associated neurotoxicity [21], [22]. Because astrocytes make up only a fraction of the cells in our MNCs, we prepared cultures of purified murine astrocytes to investigate the ability of HSV-stimulated microglial to initiate an astrocyte-specific antioxidant response. Using these cultures, we found that, in contrast to MNCs, purified mouse astrocytes exposed to virus-activated microglia (1∶5 microglia to astrocyte ratio) showed a significant induction of HO-1 (p≤0.0001; ANOVA single factor with PLSD Post-hoc analysis) and Gpx1 (p≤0.0001) mRNA by 48 h post-incubation (Figure 3B). At 48 h post-incubation, Nrf2 mRNA expression was elevated compared to astrocyte only and unstimulated microglia controls, but did not reach statistical significance.

**Figure 4:**
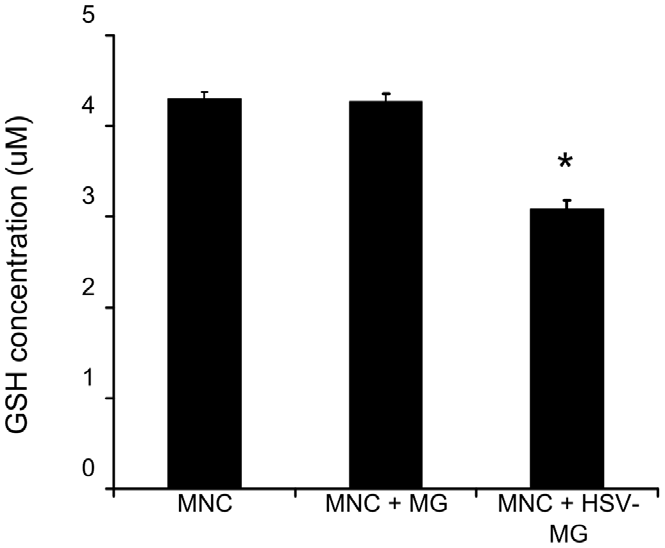
Reduced GSH production in MNCs exposed to HSV-stimulated microglia.

A battery of antioxidant enzymes are activated in response to oxidative stress including glutamate cysteine ligase (GCL), the rate limiting enzyme of glutatione biosynthesis [23]. GCL is composed of a catalytic (GCLC) and modifier (GCLM) subunit, of which the expression of GCLM is regulated by Nrf2. Recent studies report astrocyte generated GSH is important for neuronal protection during excitotoxicity and some neurodegenerative diseases [21], [22]. Therefore, we investigated the effect of HSV-infected microglia on GSH production in MNCs. We found a reduction of total GSH concentration in MNC 48 h following the addition of HSV-stimulated microglia (p≤0.0001; ANOVA single factor with a PLSD Post-hoc analysis)(Figure 4). HSV-stimulated microglia did not affect GCLM mRNA expression in MNC or astrocytes.

### Sulforaphane protects MNC from HSV-stimulated microglial toxicity

**Figure 5:**
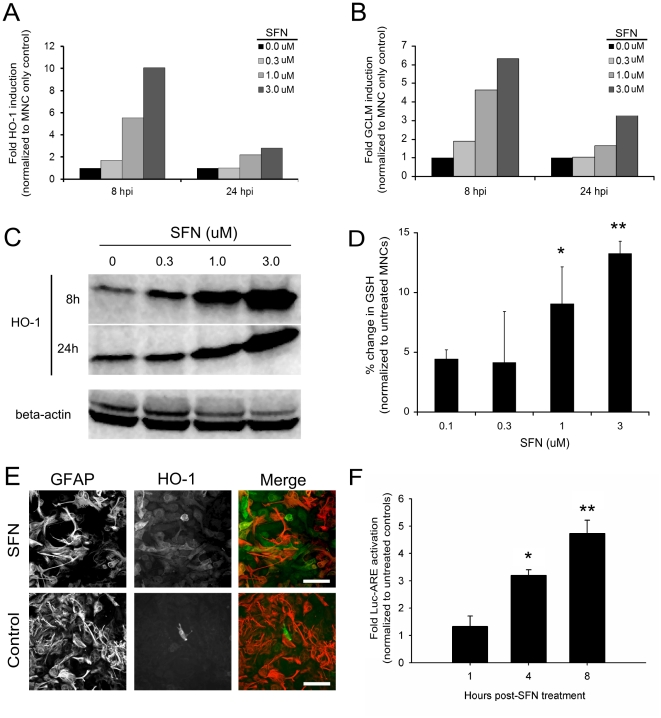
Sulforaphane elevates HO-1 expression via Nrf2/ARE activation in astrocytes. SFN (0, 0.3, 1.0 or 3.0 µM) was added onto MNC for 8 & 24 h prior to collection for semi-quantitative RT-PCR (**A & B**) and Western blot (**C**) analysis. RT-PCR of HO-1 (**A**) and GCLM (**B**) mRNA expression in MNCs following 8 & 24 h exposure to SFN. Data are presented as fold induction over saline controls and are representative of 3 separate experiments. (**C**) Western blot analysis of HO-1 protein expression in MNCs following 8 & 24 h exposure to SFN. (**D**) GSH concentration in MNCs following 48 h exposure to SFN (p≤0.0001). (**E**) SFN treated MNC (24 h) stained for HO-1 (green) and GFAP (red). Scale = 50 µm. (**F**) ARE-Luciferase reporter assay in purified astrocyte cultures following 4 and 8 h SFN treatment. *p = 0.007; **p = 0.006. Statistical significance was tested using an ANOVA single factor with PLSD Post-hoc analysis (StatView 5).

In the following studies, we investigated the efficacy of SFN, a potent stimulator of antioxidants, in protecting MNCs against the neurotoxic effects of virally-stimulated microglial cell-produced ROS. We verified the ability of SFN to induce a robust anti-oxidative stress response in MNCs by RT-PCR and Western blot. Expression analysis of MNC mRNA showed a dose-dependent increase in gene transcription of two Nrf2-regulated antioxidant proteins, HO-1 (Figure 5A) and GCLM (Figure 5B) following an 8 and 24 h SFN exposure. HO-1 protein expression was dose-dependently elevated at 8 and 24 h post-SFN treatment (Figure 5C). These data were confirmed via immunocytochemistry which detected elevated levels of HO-1 in SFN (3 µM) treated MNCs compared to untreated controls (Figure 5E). Similarly, 24 h SFN treatment resulted in a dose-dependent increase in MNC GSH (0.1 µM SFN, p = 0.0295; 0.3 µM SFN, p = 0.0029) biosynthesis confirming the functional output of SFN-induced expression of GCLM (Figure 5D). Interestingly, we found that the SFN antioxidant response was primarily in astrocytes as shown via colocalization of HO-1 positive cells in MNCs with glial fibrillary acidic protein (GFAP), an astrocyte-specific marker (Figure 5E).

SFN is known to act through the Nrf2/ARE pathway to initiate the robust production of phase II antioxidants. To confirm our finding that SFN increases HO-1 expression in mouse astrocytes, we verified that SFN treatment initiated antioxidant production through the activation of the Nrf2/ARE pathway. To this end, we transduced purified mouse astrocytes with an ARE-Luciferase lentivirus reporter system, where the lentivirus reporter is active in transduced cells only upon the binding of Nrf2 to the ARE upstream of luciferase. Using this assay, we found that 3 µM SFN resulted in the activation of Nrf2/ARE-dependent transcription at 4 h (p = 0.007) and 8 h (p = 0.006) post-treatment when compared to untreated ARE-Luciferase expressing astrocytes (Figure 5F).

To investigate the protective capability of SFN on microglial cell-induced neurotoxicity we pretreated MNC derived from β-actin promoter-luciferase transgenic Balb/c mice with SFN for 24 h prior to adding HSV-stimulated microglial cells at a 1∶5 microglia to neuron ratio. Because β-actin promoter-luciferase transgenic mice ubiquitously express the luciferase enzyme, in these studies, reduced luciferin intensity was indicative of an increase in neural toxicity. Similar to our previously published data [3], addition of HSV-stimulated microglial cells onto MNC resulted in a 40% reduction in luciferase activity indicating significant neurotoxicity. SFN pretreatment (3.0 µM) completely attenuated virus-stimulated microglial toxicity while lower concentrations (0.1, 0.3, 1.0 µM) of SFN resulted in a dose-dependent decrease in toxicity (Figure 5A). Immunohistochemical staining for MAP-2, which is specifically localized to neuronal dendrites, confirmed neuronal damage was blunted by SFN (Figure 5B). Neurotoxicity associated with virus-stimulated microglia appears to primarily affect the integrity of dendritic processes since HSV-stimulated microglia only mildly (∼15%) decreased neuronal survival. Moreover, SFN pretreatment did not dramatically increase neuronal survival in cultures with HSV-stimulated microglia. Neuronal survival was determined by assessing the number of cells double-labeled with NeuN, an neuron-specific antibody, and the nuclear intercolating dye, Hoechst 33342.

### Systemic sulforaphane injections reduce brain inflammation and ROS production *in vivo*

**Figure 6:**
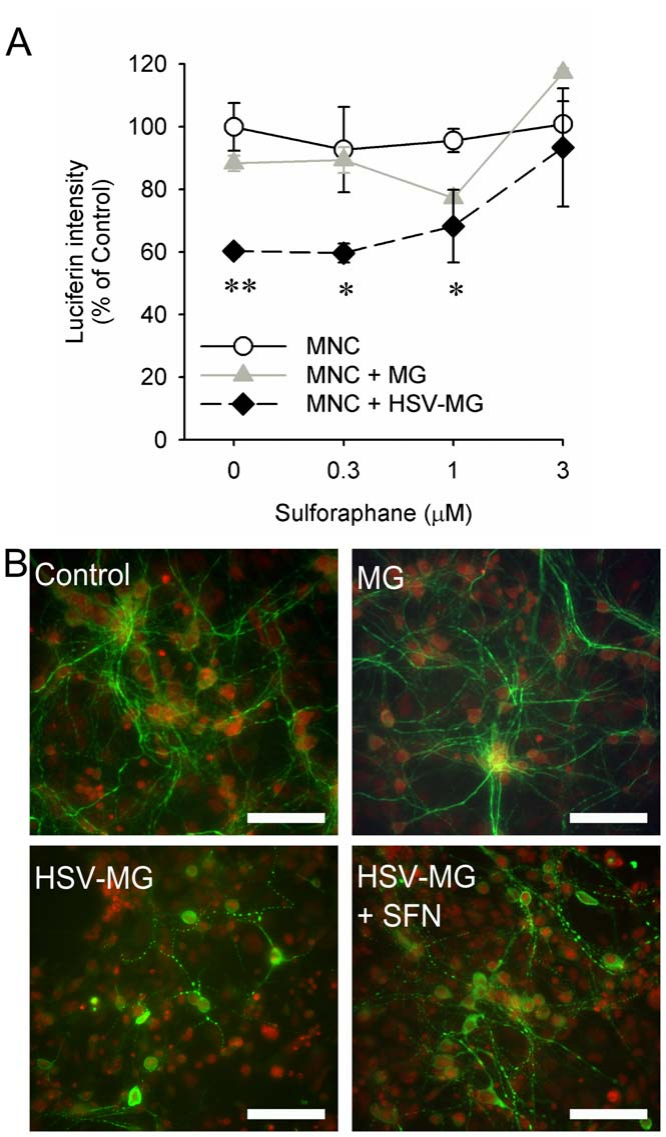
Sulforaphane protects mixed neural cultures from HSV-stimulated microglial neurotoxicity. Mixed neural cultures from β-actin luciferase Balb/c mice were pretreated for 24 h with SFN (0, 0.3, 1.0 and 3.0 µM). Purified murine microglia from Balb/c mice (non-luciferase expressing) were infected with HSV-1 at 2 MOI for 90 min (+HSV−Mg) and added at a 1 to 5 microglia: neural cell ratio. Cultures were maintained for 48 h before analysis of neurotoxicity via (**A**) luminescence or (**B**) immunohistochemistry. Unstimulated microglia (+Mg) and MNC or astrocyte only cultures (C) were used as controls. (**A**) Data are presented as mean luciferin intensity (% of MNC only control) ± SD. These data are representative of 3 separate experiments. *p≤0.1; **p≤0.05. (**B**) MAP-2 immunohistochemistry (green) in MNCs alone (Control), MNCs with unstimulated microglia (MG), and MNCs with HSV-stimulated microglia alone (HSV−MG) or following 3 µm SFN treatment (HSV−MG+HSV). Cells counterstained with propidium iodide (red). Scale = 50 µm.

We next tested the effect of intraperitoneal (i.p.) SFN injection on brain inflammation and ROS production associated with herpes encephalitis. To minimize the effect of SFN on viral entry into the brain, mice were intranasally infected and allowed to establish HSV-1 infection for 3 days prior to receiving SFN treatments. SFN-treated mice received i.p. injections of 50 mg/kg SFN for 4 consecutive days (3–6 d p.i.) while control mice received equal volumes of i.p. saline. RT-PCR detection of HSV glycoprotein D (GlyD) expression, a viral protein indicative of active viral replication, showed equal amounts of virus in SFN and control treated mice, demonstrating that systemic SFN did not impact viral entry into the brains of HSV-infected mice (Figure 6).

**Figure 7:**
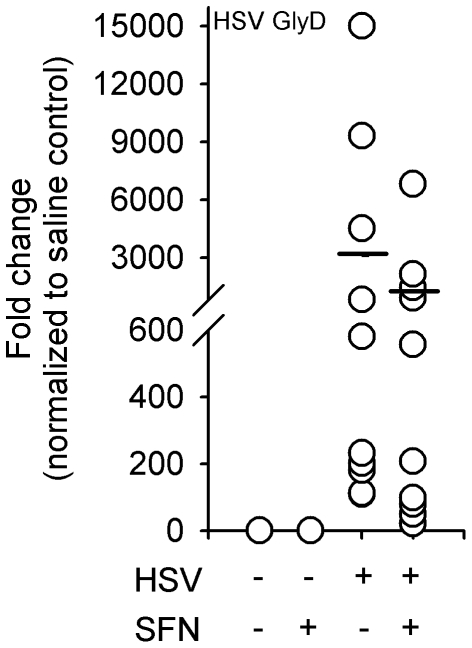
Sulforaphane does not affect establishment of HSV brain infection. Balb/c mice were infected with HSV-1 and given four 100 µl doses of SFN (50 mg/kg) or saline between 3–6 d p.i.. At 7 d p.i. subcortical tissue was collected, mRNA extracted and cDNA synthesized for semi-quantitative RT-PCR of HSV-1 glycoprotein D expression.

Flow cytometry using CD11b and CD45 antibodies found that systemic SFN resulted in a ∼50% reduction in CD11b^+^, CD45^hi^ macrophage/neutrophil infiltration (11.4%) compared to saline-treated HSV-1 infected mice (22.8%) (Figure 7A). The primary mode for macrophages to clear a pathogen is through ROS production [24], therefore we quantified intracellular ROS in CD11b^+^, CD45^hi^ cells using DCFH-DA (Figure 7B). We found that, in addition to a reduction in total CD11b^+^, CD45^hi^ infiltrate, a smaller percentage of infiltrating macrophage/neutrophils in SFN treated HSV-1 mice (59%) were producing high levels of intracellular ROS compared to saline controls (73%).

Microglia are the primary responders during viral brain infection, producing robust amounts of cytokines and chemokines to recruit peripheral immune cells and combat infection. Using flow cytometric staining for MHCII, a surface protein expressed on activated, CD11b^+^, CD45^int^ microglia, we investigated whether SFN treatment affected the functional state of microglial cells in the brain during HSV-1 infection (Figure 7C). As expected, viral infection increased the percentage of activated microglia (33% MCHII+) [16]. In contrast, in SFN-treated HSV-1 mice 21% of microglia had MHCII upregulated. Injection of SFN into control mice had no effect on microglial MHCII expression.

## Discussion

Herpes virus brain infection results in devastating encephalitis. While drug therapies that inhibit viral replication have succeeded in reducing mortality, fewer than 20% of patients surviving herpes encephalitis recover without significant long-term neuropathological manifestations [1], [20]. The host's innate immune system is pivotal in mounting the initial, robust immune response during viral brain infection and includes the activation of resident microglia as well as the recruitment of peripheral macrophages and neutrophils. It is becoming increasingly clear that these cells are also liable for damage to crucial bystander tissue that is secondary to the primary infection. Indeed, ROS production by microglial cells is at least partially responsible for the neurotoxicity documented during and subsequent to herpes encephalitis [3], [4]. In this paper we show that boosting the antioxidant capacity of brain cells, using SFN, can protect cultured neurons from cytotoxic ROS-producing microglia, as well as reduce *in vivo* ROS and inflammation in the brains of herpes encephalitic mice.

*In vivo* quantification of ROS generation is inherently difficult due the transient nature of free radical kinetics, thus, prior to the current study, measure of oxidative stress in the brain was based on the accumulation of free radical-mediated tissue damage during HSV-1 infection [3]. In this paper, we combined the sensitivity of the ROS indicator, DCFH-DA, with the isolation of brain leukocytes to successfully quantify intracellular free radical production in specific immune cell populations during viral infection. As previously reported [5], we confirm that HSV infection resulted in a robust increase in brain CD11b^+^, CD45^hi^ leukocytes at 7 d p.i.. We extended these observations to show that brain macrophages/neutrophils in herpes encephalitic mice significantly increased their production of ROS compared to controls (Figure 1). These data indicate that viral brain infection results in a ROS-rich brain, a potentially toxic environment for crucial neuronal cells. Furthermore, we report that elevated ROS is due to both the increased number of infiltrating, ROS-producing leukocytes as well as the upregulation of intracellular ROS production at the level of the individual cell.

The antioxidative stress response is crucial in preventing damage to bystander cells during ROS-mediated pathogen clearance. Our previous studies showed that increased luminescence in the brain of HO-1 promoter-luciferase expressing transgenic mice during HSV-1 infection was indicative of a counteractive antioxidative stress response being initiated [5]. Semi-quantitiative RT-PCR analysis of HO-1 performed in this study confirms these previous findings and provides evidence of a more extensive antioxidative response, including the recruitment of Gpx1 (Figure 2). Interestingly, Nrf2 mRNA expression did not increase in infected mice despite an increase in its downstream transcriptional targets, HO-1 and Gpx1. Activation of Nrf2 is dependent on its dissociation from the cytosol sequestering/degradation-targeting Kelch-like ECH-associating protein 1 (Keap1) [25]. It is likely that, at rest, sufficient cellular Nrf2 protein is available in the cytosol and that the Nrf2/ARE antioxidant pathway is triggered not by increased expression of Nrf2, but by the translocation of existing Nrf2 into the nucleus. Nonetheless, although present, the antioxidant response in the brains of encephalitic mice is insufficient to perturb the high mortality and accumulation of oxidative damage that is characteristic of HSV-1 brain infection [15], [16].

Oxidative stress insults have been shown to trigger antioxidant responses in neurons, astrocytes and microglial cells [26], [27], [28]. Neurons, while capable of implementing an antioxidant response, are still highly susceptible to ROS because of a relatively low level of resting glutathione and poor upregulation of antioxidant enzymes [29], [30]. We found that MNCs, consisting largely of neurons (85–90% neurons, 10–15% astrocytes, <2% microglia), fail to upregulate key antioxidants, HO-1 and Gpx1, upon exposure to HSV-stimulated microglia (Figure 3). Similarly, HSV-stimulated microglia reduced GSH levels in MNCs (Figure 4). MNCs also contain astrocytes, which are thought to be the primary source of antioxidant production in the brain [21], [22]. In support of this notion, we found that purified murine astrocytes respond to HSV-stimulated microglia by increasing expression of HO-1, Gpx-1 and Nrf2 mRNA (Figure 3), indicating that the lack of detectable astrocyte-associated HO-1 and Gpx1 upregulation in MNCs was not due to the astrocyte's inability to produce the antioxidants but rather their low overall number in the MNC system. Based on the delayed upregulation of astrocyte antioxidants (≥48 h), the low percentage of astrocytes in MNC (10–15%) and the rapid production of ROS in HSV-1 (∼3 hpi) stimulated microglia, we conclude that the antioxidant response in MNC is insufficient to combat the robust free radical production and subsequent neurotoxicity mediated by virus-exposed microglia.

Antioxidant enhancement is a promising therapeutic strategy for modulating brain inflammation and associated neurotoxicity [31], [32]. Sulforaphane enhances cellular antioxidant levels by activation of the Nrf2/ARE pathway, resulting in the upregulation of phase II antioxidant enzymes HO-1 and Gpx1 [12], both of which have been found to have neuroprotective actions during brain inflammation [32] [13], [20]. SFN has been found to confer neuroprotection in animal models of Parkinson's Disease, ischemia, traumatic brain injury and glutamate excitotoxicity [7], [13], [33], [34], [35] suggesting its potential use as a treatment for oxidative stress during HSV-encephalitis. In this study, we demonstrate that SFN promotes an antioxidant response in MNC (Figure 5). This response appears to derive primarily from the astrocytes within the MNC (Figure 5E) and occurs via activation of the Nrf2/ARE complex (Figure 5F). Indeed, Nrf2-mediated antioxidant upregulation in astrocytes is thought to be the primary neuroprotective mechanism in the brain against ROS-induced neurotoxicity [36], [37]. Most important was our observation that SFN pretreatment conferred significant protection to neurons in MNCs from exposure to HSV-stimulated microglia (Figure 6). These data implicate SFN as a candidate therapeutic for viral infection-induced brain neurotoxicity. Although, HO-1, Gpx1 and GSH were used in this study as indicators of SFN action in the brain, these studies are not meant to imply that SFN's neuroprotective effects are fully mediated by a single antioxidant. Indeed, studies have shown that overexpression of single antioxidants provides insufficient neuroprotection in comparison to compounds that simultaneously upregulate multiple antioxidants [38].

**Figure 8:**
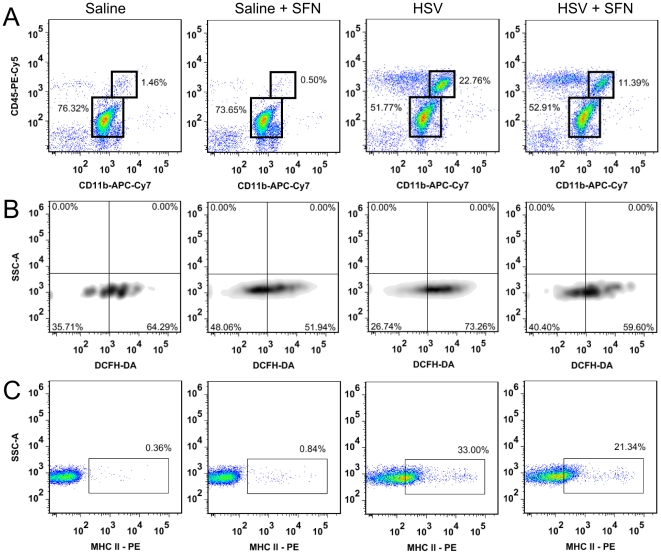
Systemic sulforaphane reduces macrophage infiltration, macrophage-specific ROS, and microglial activation in the brains of herpes encephalitic mice. Balb/c mice were infected with HSV-1 and given four 100 µl doses of SFN (50 mg/kg) or saline between 3–6 d p.i.. At 7 d p.i. whole brain leukocytes were isolated and stained for flow cytometry. (**A**) Flow cytometry plots of CD45 and CD11b staining in both uninfected and HSV-infected mice with or without SFN treatment. CD11b^+^, CD45^hi^ macrophages and neutrophils are highlighted in the upper box and microglial cells (CD11b^+^, CD45^int^) in the lower box. (**B**) CD11b^+^, CD45^hi^ macrophages/neutrophils were gated on for DCFH-DA analysis of ROS production. Cells were subdivided into high (bottom right quadrant) and low (bottom left quadrant) DCFH-DA expressing groups. (**C**) The percentage of activated microglial cells activation was assessed in both uninfected and HSV-infected mice with or without SFN treatment by detection of MHCII expression on CD11b^+^, CD45^int^ cells.

Based on our *in vitro* data, the ability of SFN to cross the blood-brain barrier [12], and evidence that systemic SFN increases brain antioxidant gene expression, decreases microglial activation, and blunts neutrophil infiltration [12], [39], we investigated the effect of systemic SFN on the inflammatory response triggered during herpes encephalitis. We found that SFN treatment significantly decreased ROS production in CD11b^+^, CD45^hi^ macrophage/neutrophils when compared to untreated HSV-infected mice (Figure 8B). In addition to reduced leukocyte-produced ROS, a more profound anti-inflammatory profile was observed in the brain of SFN-treated encephalitic mice. We found that SFN treatments significantly decreased microglial cell activation (Figure 8C) as well as reduced the number of brain-infiltrating macrophages/neutrophils (Figure 8A). Microglial cells, which normally do not express MHCII but upregulate the activation marker by exposure to interferon-γ [40], were shown to express significantly less MHCII in HSV-infected mice treated with SFN. Taken together with our observation that SFN reduced macrophage/neutrophil-produced ROS, these data indicate a less inflamed and less ROS rich brain environment upon systemic infusion of SFN, supporting its therapeutic potential during viral encephalitis.

Despite reports in the literature [13], [41], our regiment of systemic SFN treatment did not markedly increase brain HO-1 expression in control, uninfected mice. Repeated administration of SFN *in vivo* is likely to attenuate HO-1 production, a phenomenon reported in cultured astrocytes [42], as a negative feedback mechanism to prevent excess antioxidant expression. The effects of SFN in the brain may be kept quiescent until presentation of an oxidative stress insult (i.e., viral brain infection), during which its anti-inflammatory actions are required. In our model, intranasal HSV infection alone induced robust HO-1 expression in the brain (Figure 2), such that a significant difference in HO-1 expression in HSV-infected, SFN-treated mice was not detectable. Future experiment are needed to examine the kinetics of SFN action in the brain and determine whether its beneficial actions are due to its ability to mount a quicker or more prolonged antioxidant response, rather than a simple correlation with the level of antioxidant expression in the brain,

The more generalized anti-inflammatory affect of SFN on the HSV-infected brain does not appear to be a result of SFN affecting viral entry into the brain because a similar amount of viral expression was observed in control untreated encephalitic mice (Figure 7). Rather, it is likely a secondary effect of the SFN-mediated reduction in macrophage ROS production. Our previous work showed that ROS production drives synthesis of cytokines and chemokines in HSV-stimulated murine microglia [4]. Similarly, inhibition of NADPH oxidase, the primary cellular manufacturer of the superoxide free radical, has been shown to reduce the production of cytokine/chemokines in macrophages and decrease macrophage-mediated neurotoxicity in response to HIV-tat stimulation [43]. Thus, reduced ROS production in macrophages initially recruited into the brain during HSV-1 infection may result in their blunted production of cytokines and chemokines. Reduced levels of essential proinflammatory mediators such as interferon-γ and macrophage chemoattractant protein-1, would subsequently blunt the continued recruitment of peripheral macrophages as well as the activation of resident microglial cells [14], [44].

Due to the aggressive replication and lytic nature of HSV-1 infection, it is unlikely that prophylactic SFN would protect against fatal herpes encephalitis in the absence of antiviral therapy. Indeed, we found equal mortality outcomes in both saline and SFN-treated HSV-infected mice. However, post-encephalitic mice continue to have active and persistent brain inflammation which is associated with manifestation of severe cognitive deficits [15]. Data presented in this manuscript support the notion that patients may benefit from SFN treatments used in combination with antiviral therapy during active herpes encephalitis, as well as when given during post-encephalitic recovery.

## Materials and Methods

### Ethical Statement

The animal use protocols used were approved by the University of Minnesota Institutional Animal Care and Use Committee (Protocol Number: 1105A99494).

### Virus

HSV-1 strain 17 syn+ was propagated and titrated using a plaque assay on rabbit skin fibroblasts (CCL68; American Type Culture Collection, Manassas, VA).

### Intranasal HSV-1 infection

Eight to ten-week-old female Balb/c mice (Charles River Laboratories™, Boston, MA, USA) were infected via intranasal administration with 1.0×10^5^ HSV-1 plaque-forming units (PFU)/nostril (2.0×10^5^ PFU/mouse). At 7 d p.i., mice were anaesthetized with ketamine/xylazine, decapitated and brains dissected for flow cytometric analysis or RNA extraction.

### Sulforaphane treatments

R,S-Sulforaphane was purchased form LKT Laboratories (St. Paul, MN) and added to mixed neural cultures or purified astrocyte cultures at 0, 0.3, 1.0, 3.0 µM. Systemic sulforaphane was administered at 50 mg/kg.

### Isolation of brain leukocytes and flow cytometry

Leukocytes were isolated from HSV-infected murine brains using a previously described procedure [45], [46]. Briefly, brain tissues harvested from 4 to 6 animals were minced finely in RPMI (2 g/L d-glucose and 10 mM HEPES) and mechanically disrupted (in Ca/Mg free HBSS) at room temperature for 20 min. Single cell preparations from infected brains were resuspended in 30% Percoll and banded on a 70% Percoll cushion at 900 g at 15°C. Brain leukocytes obtained from the 30% to 70% Percoll interface were stained with anti-mouse immune cell surface markers for 45 min at 4°C (CD45-Allophycocyanin (APC), CD11b-APC-CY7, MHC Class II-phycoerythrin (PE); BD Biosciences, San Jose, CA) and analyzed by flow cytometry using a BD FACSCanto. Live leukocytes were gated using forward scatter and side scatter parameters and analyzed using FlowJo software (TreeStar, Inc.). The production of intracellular ROS was measured by the oxidation of 2′,7′-Dichlorofluorescin diacetate (DCFH-DA; Sigma; St. Louis, MO).

### Real Time PCR

Real-time PCR was performed on both cultured cells and fresh tissue. Cell culture extracts were collected and RNA purified using a RNAse Easy Kit (Qiagen). RNA was extracted from murine brains using a 2-mm-thick section of subcortical (bregma ∼4.0 mm). The fragment was trimmed of excess cortical tissue and the remaining subcortex assessed for mRNA expression of anti-oxidative enzyme mRNA. Total RNA was extracted from brain tissue homogenates using the TRIzol Reagent (Invitrogen, Carlsbad, CA) according to the manufacturer's instructions. For both *in vitro* and *in vivo* collected RNA, cDNA was synthesized using 1 to 5 µg of total RNA, SuperScript II reverse transcriptase (Invitrogen Life Technologies, Carlsbad, CA) and oligo dT_6–12_ primers (Sigma-Genosys, The Woodlands, TX). Semi-quantitative real-time PCR was performed using the FullVelocity SYBR Green QPCR master mix (Stratagene, La Jolla, CA). The 25 µl final reaction volume consisted of premade reaction mix (SYBR Green I dye, reaction buffer, Taq DNA polymerase, and dNTPs), 0.3 mM of each primer, and 0.5 ng cDNA in water. Reaction conditions for PCR for the Mx3000P QPCR System (Stratagene) were as follows: polymerase activation at 95°C for 5 min, 40 denaturation cycles of 95°C for 10 s, and annealing/elongation at 60°C for 30 s. The relative product levels were quantified using the 2(-Delta Delta C(T)) method [47] and were normalized to the housekeeping gene hypoxanthine phosphoribosyl transferase-encoding (HPRT; NM_013556). Forward and reverse primer sequences were designed from the murine genes of: heme oxygenase-1 (HO-1; NM_010442): 5′-cacgcatatacccgctacct-3′ and 5′-ccagagtgttcattcgagca-3′; glutathione peroxidase-1 (Gpx1; NM_008160): 5′-gtccaccgtgtatgccttct-3′ and 5′-cctcagagagacgcgacatt-3′; nuclear factor-E2-related factor 2 (Nrf2; NM_010902): 5′-atgccagccagctgacctcctta-3′ and 5′-agacggtggcagcatgccttc-3′; glutatmate cysteine ligase modifier (GCLM; NM_008129.3): 5′- ccttggagcatttacagccttact-3′ and 5′- agttctttcgggtcattgtgagtc-3′; HSV Glycoprotein D (GlyD): 5′-atccgaacgcagccccgctg-3′ and 5′-tctccgtccagtcgtttatctt-3′.

### Glutathione measurements

Total glutathione concentration was determined using the GSH-Glo(TM) Glutathione Assay Kit (Promega; Madison, WI).

### Microglial cell cultures

Murine cerebral cortical cells from 1-day-old Balb/c mice were dissociated after a 30- min trypsinization (0.25%) and were plated in 75-cm^2^ Falcon culture flasks in DMEM containing 10% heat-inactivated fetal bovine serum (FBS) and antibiotics. The medium was replenished 1 and 4 days after plating. On day 12 of culture harvested cells were plated in a 60-mm petri dish and incubated for 15 min at 37°C. After extensive washing with culture medium, adherent microglia were collected with a rubber policeman and centrifuged at 1000 rpm for 10 min. Purified microglial cell cultures were comprised of a cell population in which >98% stained positively with MAC-1 antibodies and <2% stained positively with antibodies specific to glial fibrillary acid protein, an astrocyte marker. For qPCR and neurotoxicity assays, purified murine microglia were stimulated with HSV-1 (2 MOI) for 90 min in suspension. To eliminate residual HSV-1 particles, microglia were lightly trypsinized (0.1% trypsin in hank's balanced salt solution) for 15 minutes, rinsed two times in serum free DMEM and resuspended in 7% FBS DMEM. Microglia were then added to mixed neural cultures or astrocytes at a 1∶5 microglial to neural cell ratio.

### Neural cell cultures

Separate neural cell cultures were established from wild-type Balb/c and transgenic β-actin promoter-luciferase Balb/c mice. Following dispersion of fetal (d 15 of gestation) cerebral cortices with trypsin, cells (5×10^5^ or 2×10^5^/500 ml) were plated into collagen coated wells of 24-well plates with DMEM containing 10% heat-inactivated FBS and antibiotics. On day 5, the culture medium was replaced with DMEM containing 10% heat-inactivated FBS, uridine (33.6 mg/ml), and fluorodeoxyuridine (13.6 mg/ml) to suppress glial cell growth. After 24 h, cells were replaced with DMEM containing 10% heat-inactivated FBS. Culture medium was changed every 4 days thereafter. On day 7 the neural cell cultures consisted of approximately 85–90% neurons (stained with a rabbit anti-MAP-2 antibody (Millipore; #AB5622; 5 µg/mL) and containing characteristic processes and birefringent cell bodies), 10–15% astrocytes (stained with rabbit anti-glial fibrillary acid protein antibody; Incstar, Stillwater, MN), and <2% microglia (stained with a rat anti-MAC-1 antibody; Roche Applied Science, Indianapolis, IN).

### Astrocyte cultures

Astrocytes were prepared from 1- to 2-day-old neonatal Balb/c mice. Brain tissues were dissociated and resuspended in DMEM containing penicillin (100 U/ml), streptomycin (100 µg/ml), gentamicin (50 µg/ml) and Fungizone® (250 pg/ml) and plated onto poly-L-lysine (20 µg/ml)-coated 75-cm^2^ flasks at a density of 80–100×10^6^ cells/flask and incubated at 37°C in a 6% CO2 incubator. Culture medium was changed at a weekly interval. On day 21, flasks were shaken at 180–200 rpm for 16 h followed by trypsinization with 0.25% trypsin in HBSS for 30 min. After adding FBS (final concentration 10%), centrifugation and washing, cells were seeded into new flasks with DMEM followed by medium change after 24 h. The subculture procedure was repeated four times at a weekly interval to achieve highly purified astrocyte cultures (99% of cells reacted with GFAP antibody) which were plated onto 24-well tissue culture plate (0.5 to 1×10^6^ cells/well) plates for RNA extraction.

### Immunocytochemistry

Mixed neural cultures plated onto chamber slides were fixed with 4% paraformaldehyde followed by washing with PBS and incubation with 10% normal donkey serum in PBS for 1 h at room temperature (RT). Primary antibodies for mouse anti-HO-1 (Assay Design; #OSA-110; 1∶100), rabbit anti-GFAP antibodies (1∶1000, 1 mg/ml), mouse anti-MAP-2 (Millipore; #AB5622; 5 µg/mL), or anti-NeuN antibody (Chemicon, Temecula, CA; 1∶100) were added and incubated overnight at 4°C. After washing, secondary antibody (rhodamine- or FITC-conjugate) was added for 1 h at RT followed by nuclear labeling with Hoechst 33342 (Chemicon, Temecula, CA; 1 µg/ml) and viewing under fluorescent microscope.

### Neurotoxicity assay

Purified murine microglia from wild-type Balb/c mice were stimulated with HSV-1 (2 MOI) for 90 min in suspension. To eliminate residual HSV-1 particles, microglia were lightly trypsinized (0.1% trypsin in hank's balanced salt solution) for 15 minutes, rinsed two times in serum free DMEM and resuspended in 7% FBS DMEM. Microglia were then transferred at a 1∶5 microglia to neuron ratio onto cultured neurons obtained from β-actin promoter-luciferase transgenic mice. D-Luciferin potassium salt (Gold Biotechnology; St. Louis, MO) was added to each well and luciferase activity measured on a plate reader 48 h after microglial cell transfer. MNCs were pretreated for 24 h with select concentrations (0, 0.3, 1.0, 3.0 µm) of SFN prior to transfer of microglia.

### Western Blot

Following SFN treatment cell lysates were collected, electrophorezed in 12% acrylamide/bis-acrylamide electrotransfered onto nitrocellulose membrane and probed with mouse anti-HO-1 antibody (Assay Design; #OSA-110). A rabbit anti-β-actin antibody (Cell Signaling; #4970) was used as a loading control. Alkaline phosphatase-conjugated secondary antibodies with chemiluminescence detection was used with a Kodak Image Station to capture protein band images.

### Statistical analysis

For comparison of means of multiple groups, analysis of variance (ANOVA) was performed followed by a Fisher's protected least significant difference (PLSD)-test. For comparison of means of pairs of data a two-tailed Student's T-test for paired samples was applied.
